# Neural Pattern of Chanting-Driven Intuitive Inquiry Meditation in Expert Chan Practitioners

**DOI:** 10.3390/bs15091213

**Published:** 2025-09-05

**Authors:** Kin Cheung George Lee, Hin Hung Sik, Hang Kin Leung, Bonnie Wai Yan Wu, Rui Sun, Junling Gao

**Affiliations:** 1 Centre of Buddhist Studies, The University of Hong Kong, Hong Kong SAR, China; glee123@hku.hk (K.C.G.L.); hinhung@hku.hk (H.H.S.); hank.leung@hku.hk (H.K.L.); bonniewu@hku.hk (B.W.Y.W.); 2 Department of Rehabilitation Sciences, The Hong Kong Polytechnic University, Hong Kong SAR, China; ripplesun@outlook.com

**Keywords:** Chan/Zen, EEG, neuroscience, neuroplasticity, long-term meditators

## Abstract

Background: Intuitive inquiry meditation (Can-Hua-Tou) is a unique mental practice which differs from relaxation-based practices by continuously demanding intuitive inquiry. It emphasizes the doubt-driven self-interrogation, also referred to as Chan/Zen meditation. Nonetheless, its electrophysiological signature remains poorly characterized. Methods: We recorded 128-channel EEG from 20 male Buddhist monks (5–28 years Can-Hua-Tou experience) and 18 male novice lay practitioners (<0.5 year) during three counter-balanced eyes-closed blocks: Zen inquiry meditation (ZEN), a phonological control task silently murmuring “A-B-C-D” (ABCD), and passive resting state (REST). Power spectral density was computed for alpha (8–12 Hz), beta (12–30 Hz) and gamma (30–45 Hz) bands and mapped across the scalp. Mixed-design ANOVAs and electrode-wise tests were corrected with false discovery rate (*p* < 0.05). Results: Alpha power increased globally with eyes closed, but condition- or group-specific effects did not survive FDR correction, indicating comparable relaxation in both cohorts. In contrast, monks displayed a robust beta augmentation, showing significantly higher beta over parietal-occipital leads than novices across all conditions. The most pronounced difference lay in the gamma band: monks exhibited trait-like fronto-parietal gamma elevations in all three conditions, with additional, though sub-threshold, increases during ZEN. Novices showed negligible beta or gamma modulation across tasks. No significant group × condition interaction emerged after correction, yet only experts expressed concurrent beta/gamma amplification during meditative inquiry. Conclusions: Long-term Can-Hua-Tou practice is associated with frequency-specific neural adaptations—stable high-frequency synchrony and state-dependent beta enhancement—consistent with Buddhist constructs of *citta-ekāgratā* (one-pointed concentration) and vigilance during self-inquiry. Unlike mindfulness styles that accentuate alpha/theta, Chan inquiry manifests an oscillatory profile dominated by beta–gamma dynamics, underscoring that different contemplative strategies sculpt distinct neurophysiological phenotypes. These findings advance contemplative neuroscience by linking intensive cognitive meditation to enduring high-frequency cortical synchrony. Future research integrating cross-frequency coupling analyses, source localization, and behavioral correlates of insight will further fully delineate the mechanisms underpinning this advanced contemplative expertise.

## 1. Introduction

Historically, Buddhist meditation practices encompass far more than just mindfulness of breath. Since the emergence of mindfulness, particularly mindfulness of breath, as a secularized treatment technique, an increasing variety of traditional Buddhist meditation practices have gained scientific recognition. In the current scientific understanding, meditation encompasses a diverse set of contemplative practices designed to systematically train attention, emotion, and self-referential processes, resulting in measurable changes in brain function and mental states (Tang et al., 2015; Lee et al., 2018). In recent years, electroencephalography (EEG) studies have consistently revealed that meditation induces alterations in oscillatory brain rhythms, with distinct patterns emerging based on practice type and expertise level. Meditation is commonly associated with increased power in slower frequencies, such as theta (4–8 Hz) and alpha (8–12 Hz), which are thought to reflect states of relaxed internal attention and enhanced self-regulation (Katyal & Goldin, 2021; Lee et al., 2018; Klimesch, 2012).

Among experienced practitioners, however, more complex dynamics are observed in higher-frequency bands. Trait-like increases in gamma-band (>30 Hz) activity, particularly over parieto-occipital and fronto-parietal regions, have been identified across multiple traditions as a potential marker of sustained attention, neural integration, and long-term neuroplastic adaptation (Braboszcz et al., 2017; Cahn & Polich, 2006; Thomas et al., 2024). Changes in the beta band (13–30 Hz) are also increasingly recognized, with some studies suggesting that beta power is modulated during states of active concentration and focused inquiry, potentially indexing the Buddhist psychological construct of *citta-ekāgratā*, or one-pointedness of mind (Thomas et al., 2024; Katyal & Goldin, 2021). The specific neural signatures of meditation are further shaped by the style of practice. For example, focused attention (FA) and open monitoring (OM) practices are both associated with increases in anterior theta activity, but they show different patterns of modulation in posterior theta and anterior alpha power (Lee et al., 2018). This highlights the need for research that examines specific contemplative traditions rather than treating meditation as a monolithic category.

For example, religious chanting, such as the silently chanting “Amitābha,” has received increasing research attention as a meditation practice. In a series of studies conducted by Gao and colleagues, chanting “Amitābha” was found to reduce the brain’s late-stage emotional response (LPP) to fear-inducing images. This result suggests that chanting “Amitābha” helps regulate deep emotional processing without affecting initial threat detection (Gao et al., 2020). These findings underscore how repetitive religious practices may uniquely modulate emotional reactions, potentially offering therapeutic benefits for stress and fear management.

Furthermore, chanting was shown to reduce activity in the posterior cingulate cortex and increase delta-wave power, indicating a dampening of self-focused thinking and the induction of a distinct state of focused relaxation compared to non-religious chanting or resting (Sik et al., 2024). It also activates prefrontal, parietal, and subcortical regions, including the amygdala and thalamus, suggesting that it recruits a positive emotional schema to counterbalance fear. Additionally, structural MRI revealed right-lateralized brain changes in long-term practitioners (Gao et al., 2019).

Among various meditation methods, the Chan School (禪宗, Chan zong), which emerged in sixth-century China, represents a distinct tradition that later spread across East Asia—known as Zen in Japan, Sôn in Korea, and Thiền in Vietnam (Wang, 2017). The term Chan derives from the Sanskrit Dhyāna, traditionally signifying a meditative state of equipoise, balance, and mental stability (Ma & Jorgensen, 2023). However, Chan Buddhism diverges from other schools by prioritizing direct experience and relational practice over epistemological or metaphysical discourse, aligning more closely with philosophical anthropology (Hershock, 2005). In this paper, ‘Zen’ and ‘Chan’ are used interchangeably to denote the same concept.

Zen practices take diverse forms, including dynamic dialogues with masters (*wèndá*), contemplation of public cases (*gōng’àn* or *koan*), chanting, and *Can-Hua-Tou* (參話頭)—a method of intuitive inquiry meditation (Hershock, 2005). While these techniques vary, a unifying theme in Chan is the cultivation of a beginner’s mind. This concept does not imply naive ignorance but rather a willingness to relinquish preconceptions and rigid cognitive schemas, fostering openness to direct insight (Hershock, 2005).

Central to this process is *Can-Hua-Tou*, a practice that targets the root of suffering—attachment to a fixed self-notion or cognitive self-schema—by systematically deconstructing it through doubt (Cai, 2005). Unlike passive meditation styles, *Can-Hua-Tou* actively engages the practitioner’s critical faculties, challenging the validity of deeply held beliefs about the self.

In essence, *Can-Hua-Tou* in the Chinese Chan lineage is unique for its active and cognitively demanding nature (Chattopadhyay, 2022; Gao et al., 2023). Rather than passively observing the breath or sensations, practitioners of *Can-Hua-Tou* continuously investigate a fundamental existential question (e.g., “Who is reciting the Buddha’s name?”) while cultivating a state of profound doubt and non-attachment (Chattopadhyay, 2022). The term *Hua-Tou* literally means “head of speech” or “the point at which (or beyond which) speech exhausts itself,” indicating its function as a practice that leads practitioners beyond conceptual thinking. This practice requires the mobilization of all attentional resources and the disciplined dismissal of extraneous thoughts, representing an intensive cognitive process that may uniquely modulate EEG dynamics. The cultivation of this “great doubt” is considered a critical component for achieving insight and is closely linked to one-pointedness of mind (Chattopadhyay, 2022).

While the neuroscientific literature on mindfulness and compassion meditation is extensive, the oscillatory signatures of inquiry-based traditions like *Can-Hua-Tou* remain underexplored. Based on recent findings, it is plausible that such practices would produce pronounced beta and gamma enhancements, reflecting the integration of sustained concentration, metacognitive monitoring, and heightened awareness (Braboszcz et al., 2017; Thomas et al., 2024). However, the specific topographies and functional significance of these oscillatory changes require further clarification.

The present study addresses this gap by investigating EEG spectral power in the alpha, beta, and gamma bands during Zen inquiry meditation in experienced Buddhist monks and novice practitioners. Based on the unique cognitive demands of *Can-Hua-Tou*, we hypothesized that experienced monks would exhibit: (a) significant beta-band activation during meditative inquiry, reflecting focused cognitive effort and one-pointedness; (b) robust gamma-band power, indicating heightened introspective vigilance and integrative neural processing; and (c) a distinct pattern of alpha-band modulation reflecting the balance between internalized attention and active cognitive engagement. This work aims to characterize the neural signatures of advanced inquiry meditation, thereby bridging contemporary cognitive neuroscience with Buddhist psychological theory (Chattopadhyay, 2022; Tang et al., 2015; Lee et al., 2018).

## 2. Materials and Methods

### 2.1. Participants

The participant cohort for this study is the same as that reported in a prior event-related potential (ERP) investigation (Gao et al., 2023). The experienced group consisted of 20 Buddhist monks (all male; mean age = 48 ± 13 years) with 5 to 28 years of dedicated practice in Can-Hua-Tou (Zen inquiry) meditation. The novice control group included 18 lay Buddhist practitioners (all male; mean age = 47 ± 11 years) with less than six months of experience in this specific practice. All participants were right-handed and provided written informed consent. The study protocol was approved by the relevant institutional ethics committee (EA1606043) and conducted in accordance with the Declaration of Helsinki (Gao et al., 2023).

### 2.2. Experimental Design and Procedure

The experimental design and procedure are consistent with those described by Gao et al. (2023). A 2 (Group: Monks, Novices) × 3 (Condition: ZEN, ABCD, REST) mixed-factorial design was employed. All tasks were performed in a quiet, dimly lit room with participants’ eyes closed. The three conditions were presented in a counterbalanced order to control for potential sequence effects.

ZEN (Meditation) Condition: Participants engaged in their standard practice of Can-Hua-Tou meditation, focusing on the inquiry, “Who is chanting the name of Buddha?”

ABCD (Active Control) Condition: As a non-meditative cognitive control, participants were instructed to repeatedly and softly murmur a neutral four-syllable sequence. This task was designed to match the phonological component of the ZEN condition without the element of self-inquiry.

REST Condition: Participants sat quietly with their eyes closed, providing a baseline measure of resting-state brain activity.

Each condition lasted for approximately 5–7 min. The present analysis focuses on the continuous EEG data recorded during these stable state periods.

### 2.3. EEG Data Acquisition and Preprocessing

Continuous EEG data were acquired using a 128-channel Geodesic Sensor Net (Electrical Geodesics, Inc., Eugene, OR, USA), with electrodes arranged according to the 10–5 system. The signal was sampled at 1000 Hz and later down-sampled to 250 Hz for analysis. The data were recorded with the reference electrode positioned at Cz, and electrode impedances were maintained below 30 kΩ, well within the recommended ≤50 kΩ threshold for high-input-impedance Geodesic amplifiers (Ferree et al., 2001; Gao et al., 2023).

Offline preprocessing was performed using the EEGLAB 13.6.5b toolbox (Delorme & Makeig, 2004) in MATLAB 2023a. Data from two participants in each group were unusable due to technical issues in which experiment-condition markers were not recorded or could not be retrieved. The outermost channels were removed due to excessive noise, leaving 108 channels for analysis. The data were band-pass filtered between 1 Hz and 45 Hz. Noisy channels and segments were identified by visual inspection. Noisy channels were then interpolated using spherical spline interpolation, and noisy segments were rejected. To remove ocular and muscle artifacts, Independent Component Analysis (ICA) was applied. Components clearly reflecting eye movements or electromyographic (EMG) activity were identified by visual inspection of their scalp topographies and time courses and were subsequently removed from the data. This step is critical for preventing non-neural artifacts from contaminating the high-frequency spectral analysis (Braboszcz et al., 2017). After artifact removal and channel interpolation, the cleaned EEG was re-referenced to the common average across all channels. A common-average reference approximates a neutral reference in dense-array EEG and reduces bias due to reference electrode activity.

### 2.4. Spectral and Statistical Analysis

The cleaned, continuous EEG data were segmented and saved as new datasets for each condition. For each dataset, the power spectral density (PSD) was computed using Welch’s method with a Hamming window based on the default parameters (window length 250; Fast Fourier Transform length: 250; overlap 0). To normalize the distribution, power values were log-transformed to dB. Finally, the average power was calculated for three frequency bands of interest: alpha (8–12 Hz), beta (12–30 Hz), and gamma (30–45 Hz).

Statistical analysis was performed using a two-way mixed-design ANOVA for each frequency band, with group (Monks, Novices) as the between-subjects factor and condition (ZEN, ABCD, REST) as the within-subjects factor. To correct for multiple comparisons across the 108 electrodes, the False Discovery Rate (FDR) procedure was applied, with a corrected significance threshold set at *p* < 0.05 (Benjamini & Hochberg, 1995). Topographic maps were generated to visualize the scalp distribution of significant findings.

### 2.5. Brain Connectivity Analysis

For brain connectivity analysis, we assessed functional connectivity post hoc using the weighted Phase Lag Index (wPLI), computed with a discrete prolate spheroidal sequence (DPSS) multitaper. The wPLI measures the consistency of non-zero phase lags between EEG signals while minimizing the effects of volume conduction. Effective connectivity was estimated using Partial Directed Coherence (PDC) with an MVAR order of 10, which evaluates the direction and strength of information flow between brain regions in the frequency domain. For each dataset, the continuous EEG was segmented into non-overlapping 2 s epochs. We computed Fourier coefficients per epoch using DPSS multitapers and estimated debiased wPLI with the FieldTrip toolbox by pooling all epochs (and tapers) as independent observations. This yielded a single connectivity matrix per frequency, which we then averaged across the frequency bins within each band of interest to obtain band-limited connectivity matrices.

## 3. Results

**Figure 1:**
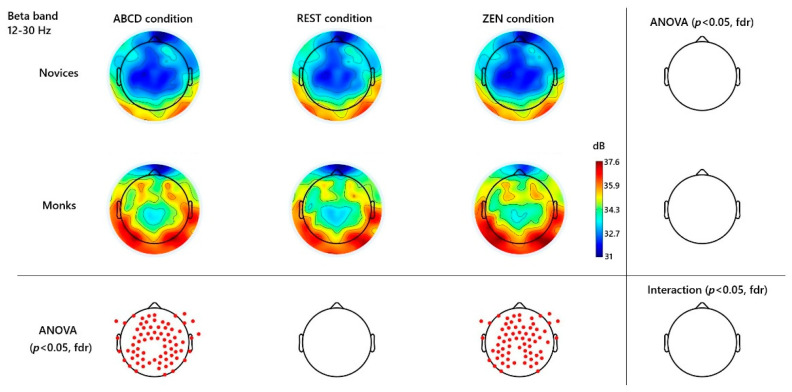
**Beta-Band (12–30 Hz) Topographies and Significant Channels.** Channels were contrasted across different groups and conditions. Red dots mark significant channels (*p* < 0.05, FDR). No significant group × condition interaction.

**Figure 2:**
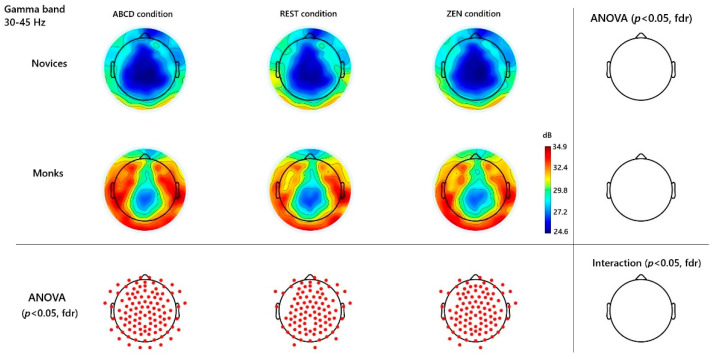
**Gamma-Band (30–45 Hz) Topographies and Significant Channels.** Channels were contrasted across different groups and conditions. Red dots mark significant channels (*p* < 0.05, FDR). No significant group × condition interaction.

**Figure 3:**
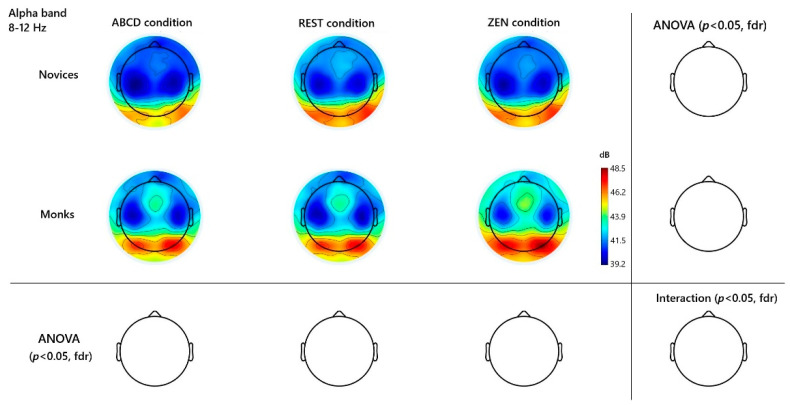
**Alpha-Band (8–12 Hz) Monks ABCD vs. ZEN Topographies and Channel Differences**. Channels were contrasted across different groups and conditions. No significance found after FDR correction.

Spectral analyses revealed frequency-specific distinctions between long-term Chan monks and novice controls. Figure 1 (beta), and Figure 2 (gamma), Figure 3 (alpha), illustrate scalp topographies and significance maps. An initial inspection of the spectral topographies (Figure 1, Figure 2 and Figure 3) revealed that the beta and gamma bands exhibited the most robust signatures of expertise in Zen inquiry meditation, whereas alpha oscillations exhibited only modest, non-significant trends. Below, we detail these findings in regions that correspond to the peaks visible in the group-averaged scalp maps.

In the beta band (12–30 Hz), the major effect occurred over the frontal and bilateral temporal lobes. Experienced monks displayed a marked elevation in beta power in conditions including the restful baseline and the active Hua-Tou inquiry state, when compared to novices (e.g., channel Fz: *t*(33) = 2.30, *p* = 0.046 FDR; channel Pz *t*(33) = 2.18, *p* = 0.054 FDR; channel behind Cz named E55: *t*(33) = 2.23, *p* = 0.049 FDR). Importantly, this beta enhancement was already present during the non-meditative ABCD control task and did not increase further during inquiry (*p* > 0.05 FDR); No difference in beta wave power was found during the rest condition. See Figure 1.

Turning to the gamma band (30–45 Hz), a complementary but trait-like pattern emerged over almost the whole brain regions. Monks maintained uniformly high gamma power across rest, ABCD, and inquiry, with all pairwise contrasts against novices surviving FDR correction (main group effect—e.g., channel Fz: *t*(33) = 3.53, *p* < 0.001 FDR; channel Pz: *t*(33) = 2.92, *p* < 0.001 FDR; and channel behind Cz named E55: *t*(33) = 4.23, *p* < 0.001 FDR). Novices exhibited consistently lower gamma in every block, underscoring the trait-like nature of the monks’ gamma enhancement. See Figure 2.

Finally, in the alpha band (8–12 Hz), both groups showed the expected eye-closure–related increase in occipital alpha (O1, O2, Oz) during rest and inquiry compared to ABCD, reflecting general relaxation. However, despite visible posterior alpha hotspots in the difference maps (see Supplement Figure S1), no channels reached significance after FDR correction. Thus, unlike more passive meditation styles, Hua-Tou’s cognitively active inquiry does not reliably recruit strong alpha augmentation once multiple comparisons are taken into account. See Figure 3.

For the post hoc brain connectivity analysis, we did not find significant brain network connectivity difference in all frequency bands between the groups.

## 4. Discussion

Zen (Chan) inquiry meditation, also known as Can-Hua-Tou, is a distinctive form of meditation primarily practiced in Southeast Asia. This approach actively engages the mind in a process of inquisitive doubt, a characteristic unique to humans, which stands in contrast to another defining aspect of the human mind: belief. Indeed, we find fast-brain wave difference between experienced monks and novices, which helps us to understand Zen from a neuroscientific perspective.

### 4.1. Beta-Band Increases and One-Pointed Concentration (Citta-Ekāgratā)

A key finding was the significant increase in beta-band power (~13–30 Hz) in monks when they engaged in the inquiry meditation, an effect not seen in novices. This result is intriguing, as beta oscillations in meditation are less frequently emphasized in the literature compared to alpha/theta or gamma. In our study, the beta power in monks rose notably over parietal and occipital regions during the meditative state (Figure 2), consistent with at least one prior report of Zen meditators showing enhanced occipital beta during deep meditation (Murata et al., 2004). What could the observed increase in beta signify in this context? Beta rhythms have been linked to active thinking, focused cognitive processing, and sustaining concentration. We propose that the observed beta increase reflects the monks’ mental effort in maintaining “one-pointedness” on the *Hua-Tou* question. In Buddhist terms, *citta-ekāgratā* (one-pointedness of mind) refers to a unification of mental faculties on a single object, suppressing distractions and “hindrances” (Wallace, 2006). The monks in this study, with years of training, can enter a state of unwavering focus on the doubt or question, likely engaging in a highly active mental state rather than a quiescent one. A recent study supports this finding; advanced Tibetan monks demonstrate a distinctive high-amplitude beta wave peak during deep concentration (Neri et al., 2024). In their findings, several EEG sessions during meditation exhibited a significant and sharp peak (“bump”) in the beta frequency range (~14–30 Hz), sometimes increasing by as much as 6 dB above baseline.

In our study, beta elevation likely indexes sustained concentration, engaging attention-related parietal networks and possibly visual imagery or inner speech. That it occurred mainly in monks suggests a threshold of meditative depth is needed to elicit this response; novices may have struggled to keep the question in mind. Notably, beta remained high during later meditation periods, echoing findings that deeper absorption correlates with higher beta (Aftanas & Golocheikine, 2001). These beta effects appear to mark samādhi, as active maintenance of the meditative object. Future EEG studies combining task probes or subjective reports during Can-Hua-Tou practice could clarify how beta relates to one-pointedness or insight moments.

### 4.2. Gamma-Band Elevations and Introspective Vigilance

The most robust difference between monks and novices was observed in the gamma band (30–45 Hz), across wide swaths of the scalp, irrespective of condition. This pattern mirrors findings in long-term meditators, where both state and trait gamma elevations have been observed (Lutz et al., 2004). In our data, monks showed a gamma-rich profile even at rest, with only small additional increases during inquiry. Such ubiquity suggests that training induces enduring cortical changes supporting gamma oscillations, which are linked to large-scale neural synchronization, cognitive integration, and conscious attention. Gamma activity is often linked to neural synchronization of distributed networks, cognitive integration, and conscious attention.

In Can-Hua-Tou meditation, we interpret gamma as reflecting sustained introspective vigilance—continuous readiness and integration of cognitive resources while probing the self. The practice is described as “engaging all cognitive resources” (打成一片) in inquiry, dissolving dualities so all meditative objects are treated equally. This non-dualistic stance is echoed in Chan texts (e.g., Treasure Book of the Lotus Sect, Blue Cliff Record), which emphasize unifying mind and practice. Such mental integration may manifest in the broad fronto-parietal gamma we observed, engaging executive and attentional networks for meta-cognitive monitoring.

For example, the Treasure Book of the Lotus Sect in Lushan (廬山蓮宗寶鑑・念佛正宗) states, “Through unwavering practice, thoughts fuse into oneness… Reciting the Buddha is reciting the mind—mind and Buddha are non-dual. When the mind is non-dual, all Buddhas are one.” Similarly, the Blue Cliff Record (佛果圜悟禪師碧巖錄) notes, “Fused into oneness... Sometimes calling heaven earth, sometimes earth heaven... A serene equanimity dissolves all distinctions.” Both emphasize a fluid, non-dual mind that transcends conceptual labels, so that “ABCD” and “Buddha” are approached with the same undifferentiated inquiry.

Interestingly, gamma activity has also been associated with affective arousal and motivation; the act of generating a profound doubt might carry a certain emotional or motivational intensity (sometimes called “Great Doubt, Great Determination” in Zen training) which could further drive gamma oscillations. Our gamma findings match this description: monks displayed high gamma not only during Zen but also in ABCD and Rest, indicating a trait-like state of continuous cognitive engagement. Unlike compassion meditation in Lutz et al. (2004), where gamma rose prominently during the practice block, here gamma remained elevated across all conditions—suggesting that seasoned Can-Hua-Tou practitioners sustain active, effortful inquiry as an ongoing mental habit. This constant activation implies the fronto-parietal attention and meta-cognitive monitoring networks are always “on,” ensuring the question remains present and irrelevant thoughts are discarded.

Another aspect to consider is the possible role of emotional or motivational factors. Although Zen inquiry is primarily cognitive, it is sometimes described that one must have strong determination and even a feeling of urgency or existential concern while meditating on the question (this fervor drives the inquiry until a breakthrough). Such heightened arousal could contribute to gamma-band activation, as high-frequency oscillations have been observed during intensely compassionate or devotion-based meditations that carry strong affective components (Ospina et al., 2007; Fell et al., 2010). Although affective arousal can elevate gamma, our paradigm lacked explicit emotional induction, making attentional–introspective synergy the more likely driver. The elevated baseline gamma may also indicate training-induced plasticity or altered excitatory–inhibitory balance, supporting the view that Can-Hua-Tou practice sculpts the brain toward sustained, high-level cognitive integration.

### 4.3. Alpha-Band Modulation in Experienced Zen Meditators

Alpha oscillations (8–12 Hz) are often associated with relaxed wakefulness, internal attention, and mental tranquility. Many meditation studies have reported enhanced alpha power during meditation, sometimes more so in experienced meditators (Hardt & Kamiya, 1978; Lagopoulos et al., 2009). In our paradigm, we did not predict a large alpha difference between Rest and Zen, as both are eyes-closed conditions with reduced visual input and general relaxation. Our main expectation was that both Rest and Zen would show higher alpha than the cognitively active ABCD control, with one-pointed focus in Zen being expressed primarily in higher-frequency (beta/gamma) dynamics rather than in alpha. Indeed, both monks and novices exhibited increased alpha during Rest and Zen compared to ABCD, and the Rest–Zen differences were small and non-significant. This pattern suggests that alpha in this context reflects general relaxation and internal attention rather than a specific neural marker of meditation expertise. Monastic meditators did appear to show a trend of higher alpha during the inquiry meditation than novices (suggesting deeper relaxation or inward absorption); however, this difference did not reach statistical significance once we controlled for multiple comparisons. One possible reason is that the novices also achieved a moderate alpha increase simply by sitting quietly with eyes closed and attempting to meditate. In essence, the act of closing the eyes and directing attention inward (even without extensive training) produces a substantial alpha effect in the brain (Hardt & Kamiya, 1978), leaving relatively little room for the monks to further distinguish themselves on this measure. Moreover, the unique cognitive demand of *Can-Hua-Tou* meditation—maintaining a state of doubt and intense inquiry—may prevent alpha from rising as high as it might in a more passive relaxation or breathing meditation. Alpha is inversely related to cortical information processing; a strong engagement in analytic contemplation could lead to alpha suppression even as other meditative mechanisms may increase it (Klimesch, 2012; Hölzel et al., 2011). This might explain why, in contrast to studies of mantra or breath meditation (which show robust alpha increases in experts), our inquiry-focused monks did not significantly outperform novices in alpha power. In short, alpha oscillations in this context seem to reflect a baseline meditative relaxation that both groups accessed, rather than a distinct marker of expertise. The monks’ ability to sustain meditative alpha (i.e., not have it disrupted by stray thoughts or stimuli) might be slightly better—for instance, experienced meditators are known to exhibit “alpha blocking” resistance, keeping alpha steady despite distractions (Brandmeyer & Delorme, 2020). Future investigations could explore alpha reactivity (e.g., to auditory probes) during Can-Hua-Tou meditation to see if monks indeed maintain alpha differently (as an index of non-distraction and non-attachment to sensory input).

### 4.4. Integration with Buddhist Cognitive Psychology

The neural patterns observed map onto Buddhist concepts such as non-attachment and self-processing. Inquiry meditation aims to foster non-attachment to self-concepts and all thoughts. Through long-term training, monks learn to let go of ordinary self-referential thinking, viewing thoughts and self-concepts as transient. This stance was evidenced in a companion ERP study with the same participants, where monks in an inquiry state showed reduced brain responses to their own face compared to novices (Lo & Marpaung, 2020). In our spectral results, non-attachment may help monks prevent irrelevant thoughts or ego-oriented concerns from disrupting focus, sustaining high beta/gamma without the random neural activations novices may experience. Beginners, still attached to discursive thoughts and distractions, did not display the same focused rhythm profiles. The observed trend of higher alpha activity in monks may suggest a greater sense of calmness associated with non-attachment, even though the difference was not statistically significant. Monks exhibited numerically higher alpha levels during rest and meditation, which could indicate a calmer state of mind despite engaging in active doubt.

Buddhist teachings emphasize mastery of attention (sati)—directing and sustaining attention at will and monitoring mental activity. Our results support this: monks exhibited a dynamic range, increasing beta/gamma for inquiry, while novices had a blunted pattern dominated by theta and low beta during meditation. Prior work similarly found that only experienced meditators showed significant spectral perturbation and phase-locking changes during inquiry, demonstrating the ability to “flexibly modulate ongoing cognitive processes” in a way novices could not (Fucci et al., 2018). This aligns with the view that meditation enhances cognitive control and meta-awareness. The monks’ fronto-parietal gamma likely reflects engagement of attention networks; notably, the anterior cingulate cortex (ACC) and dorsolateral prefrontal cortex (DLPFC) have been implicated in meditation expertise (Tang et al., 2015). Our scalp findings (frontal gamma, parietal beta) are broadly consistent with these activations, though we did not perform source localization.

A unique feature of Zen meditation is insight (prajñā) gained through inquisitive doubt. The ultimate aim of Can-Hua-Tou practice is to trigger insight into the true nature of mind/self. Anecdotally, insight moments during meditation have been linked to transient EEG changes, such as gamma bursts, as seen in studies of “aha” moments or koan resolution. While our study was not designed to capture a specific enlightenment moment, the overall gamma elevation in monks may reflect ongoing high-level processing and heightened consciousness that create fertile ground for insight. In Buddhist cognitive terms, the doubting process is said to break down habitual schemas and self-beliefs (Vörös, 2017), requiring a mind that is both concentrated (beta) and open/flexible (gamma) to question assumptions rather than follow old thought patterns. The combination of beta (concentration) and gamma (integration/vigilance) in our monks may represent this dual signature, enabling them to hold the doubt while monitoring arising thoughts without clinging. Over time, such practice might rewire the brain to be less self-centered and more capable of self-detachment (Lo & Marpaung, 2020). In novices, the lack of strong beta and gamma activity suggests that they did not fully engage in concentration or introspective monitoring. Instead, they may have alternated between trying to apply the technique and drifting off, which Buddhist psychology describes as states of sloth and torpor or dullness.

### 4.5. Parallels to Other Meditation Traditions

Although centered on a specific Chan method, our findings parallel results from other meditation styles. High gamma in experts has been observed in Tibetan Vajrayana practitioners during compassion meditation (Lutz et al., 2008), while increased beta and alpha in focused-attention practices has been reported in Yogic or Zen contexts (Murata et al., 2004; Stigsby et al., 2011). This suggests common neural denominators across advanced meditation: the integration of calm (alpha) and focus (beta), culminating in intense clarity (gamma). However, exact magnitudes and topographies vary by technique: mantra meditation may elicit more frontal midline theta, whereas open awareness may emphasize gamma synchrony with less beta. Across traditions, advanced practice engages a broad oscillatory spectrum, combining lower-frequency relaxation with higher-frequency attentional control and cognitive integration.

## 5. Limitations

Several limitations should be noted. First, although age-matched, our sample was modest and consisted solely of older male monks, which may limit generalizability and contribute to inter-individual variability in spectral patterns. Second, meditation was performed in a controlled laboratory/retreat environment for a limited duration; participants reported reaching only about half their usual depth of inquiry (Chan, 2019), suggesting our results may underestimate EEG effects in more prolonged or intense practice, where even larger beta/gamma differences might emerge. Third, analyses focused on stationary spectral power; future work could examine cross-frequency coupling (e.g., theta–gamma; Fell & Axmacher, 2011) and coherence to assess network-level coordination. Theta activity (4–8 Hz) was not analyzed in detail, but given its link to focused attention, its role in Can-Hua-Tou meditation warrants investigation.

## 6. Conclusions

This study provides novel evidence that Can-Hua-Tou (Zen inquiry) meditation yields distinct electrophysiological signatures distinguishing seasoned practitioners from novices. Monks demonstrated one-pointed concentration (beta) and heightened introspective vigilance (gamma) while maintaining the calm (alpha) of the meditative context. Elevated gamma across conditions suggests engagement of high-frequency networks supporting deeply integrated cognition, consistent with Buddhist descriptions of a mind both “calm and alert.”

Our findings highlight that analytical, inquiry-based meditation is not a passive state but one of sustained neuronal activation, contributing to the diversity of neural profiles across contemplative practices. The monks’ EEG patterns may represent the neural correlates of *prajñā* (wisdom) and *samādhi* (concentration), with synchronized gamma reflecting mental unity and potential transformation. Future research combining subjective reports, behavioral measures, and neuroimaging could deepen understanding of how such practices shape the mind and brain. Beyond neuroscience, these results support applying Can-Hua-Tou as a Buddhist counseling technique to help individuals question negative thoughts and emotions, fostering a non-dualistic perspective and enabling release from fixation, ultimately cultivating present-moment equanimity.

## Data Availability

The original contributions presented in this study are included in the article/Supplementary Materials. Further inquiries can be directed to the corresponding author.

## Supplementary Materials

The following supporting information can be downloaded at: https://www.mdpi.com/article/10.3390/bs15091213/s1, Figure S1. Alpha-band (8–12 Hz) topographies and channel differences for Monks: ABCD vs. ZEN condition. Dots mark channels with significant differences (uncorrected); darker dots indicate greater significance. Figure S2. Power spectra of channel Fz for all groups and conditions. Figure S3. Power spectra of a channel near Cz (E55) for all groups and conditions. Figure S4. Power spectra of channel Pz for all groups and conditions.
