# Supplementary material for: Neural Pattern of Chanting-Driven Intuitive Inquiry Meditation in Expert Chan Practitioners
Source: Behav Sci (Basel). 2025 Sep 5;15(9):1213. doi: 10.3390/bs15091213 (PMC12466501; doi:10.3390/bs15091213)
Supplement: Supplementary file 1 [file behavsci-15-01213-s001.zip › behavsci-3759998-supplementary.pdf]

Supplementary Materials

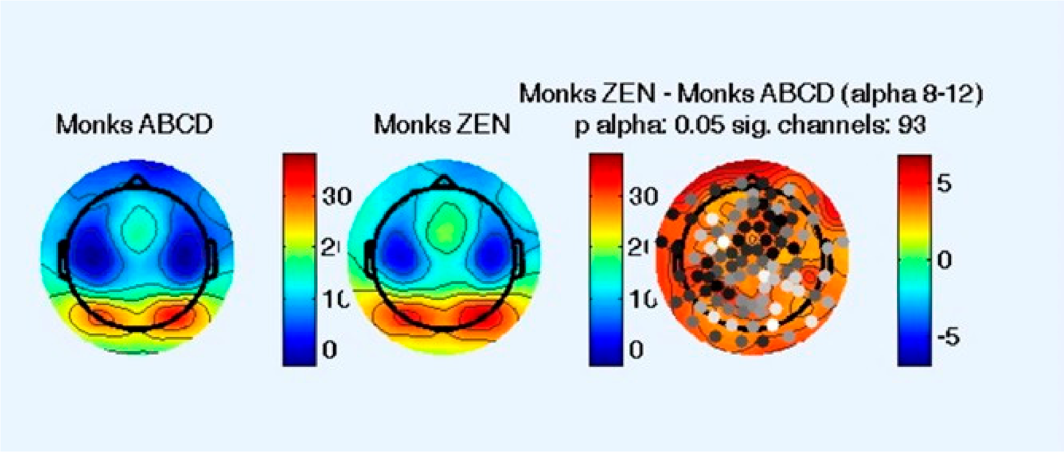

Figure S1. Alpha-band (8–12 Hz) topographies and channel differences for Monks: ABCD vs. ZEN condition. Dots mark channels with significant differences (uncorrected); darker dots indicate greater significance.

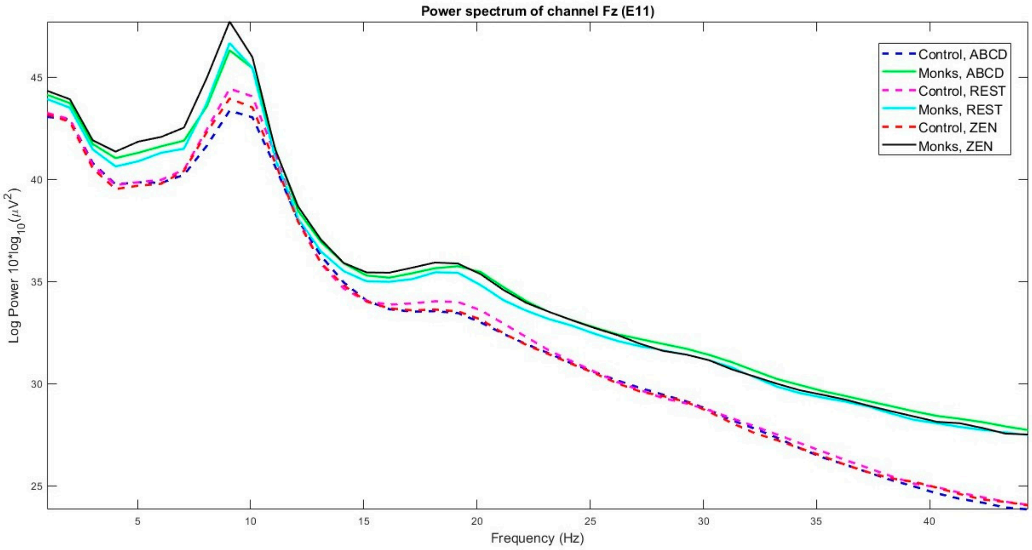

Figure S2. Power spectra of channel Fz for all groups and conditions.

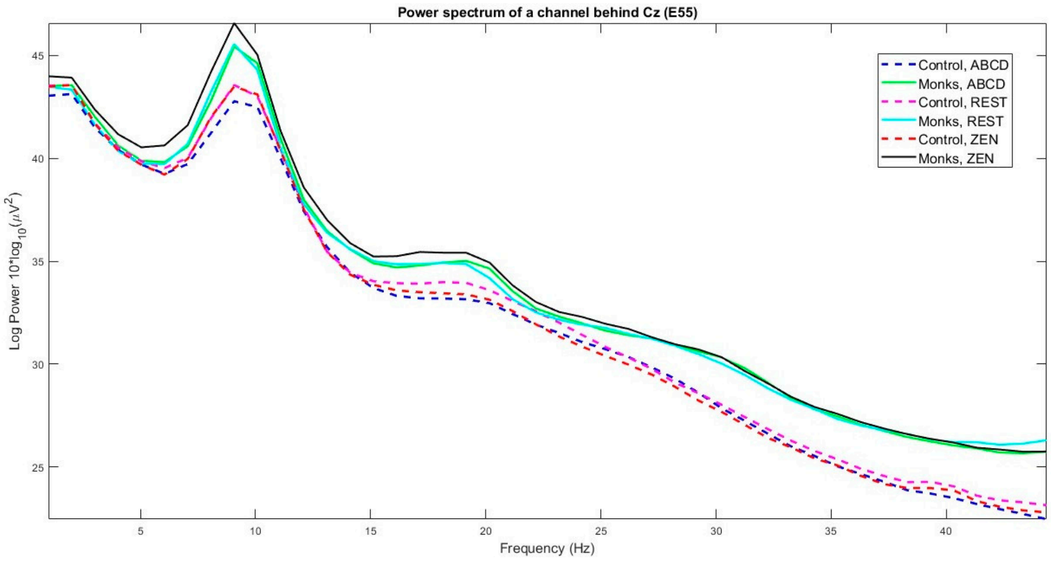

Figure S3. Power spectra of a channel near Cz (E55) for all groups and conditions.

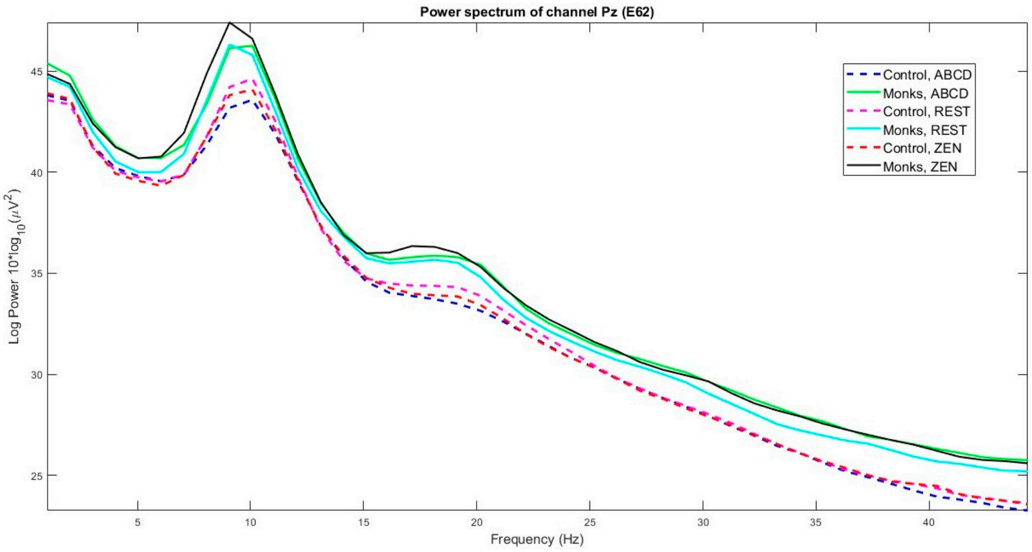

Figure S4. Power spectra of channel Pz for all groups and conditions.
